# Inhaled MgSO4 in acute asthma: are we on the right course?

**DOI:** 10.1016/j.jped.2024.08.001

**Published:** 2024-08-21

**Authors:** Sérgio Luís Amantéa

**Affiliations:** Universidade Federal de Ciências da Saúde de Porto Alegre (UFCSPA), Departamento de Pediatria, Porto Alegre, RS, Brazil

Dear Editor

I found the article entitled “Inhaled magnesium versus inhaled salbutamol in rescue treatment for moderate and severe asthma exacerbations in pediatric patients” by Debiazzi et al., published in this journal, very interesting.^1^ Although the acute management of asthma is a frequent situation in emergency departments, we don't have any new drugs capable of modifying the acute course of the disease. Our care protocols have been little changed over the last 20 years. The mainstays of pharmacological treatment remain the use of inhaled drugs (agonist beta-2 adrenergics and anticholinergics), as well as corticosteroids, used by different routes of administration.^2^

In this scenario, magnesium sulphate (MgSO_4_) has been considered as a therapeutic option in the management of acute asthma refractory to standard therapy. Both the intravenous and inhaled routes have been used; however, only the benefits of the intravenous route in promoting bronchodilation have been proven and have been increasingly incorporated into care protocols.^2^^,^^3^ In the inhalation route (nebulization), the results and evidence associated with its use are controversial. The inhalation route has the potential to offer some benefits, extrapolating data on the use of other drugs by this route: rapid onset of action and reduced incidence of side effects.^4^ Despite the established controversy, the most recent recommendations from the Global Initiative in National Asthma and the British Thoracic Society have modified their previous recommendations for the use of inhaled MgSO_4_ for the treatment of acute asthma attacks in an emergency setting. Contrary to data from previous reviews, more recent systematic reviews have not supported the benefits associated with the use of inhaled MgSO_4_ in the treatment of acute asthma in both adults and children.^2^^,^^3^^,^^5^^,^^6^

Debiazzi et al., in an elegant way, explicitly state that the results should be interpreted with caution, as we are evaluating data from a pilot study with a small number of patients.

We can generalize this discussion, as the data from all the literature is still insufficient to establish a critical and well-founded judgment. Not even the doses used for inhalation medication are standardized. The dose response relationship and frequency of administration at different ages are required to identify the exact amount of magnesium sulphate to be used during the acute asthma attack as inappropriate dosage cannot give the desired response.

Recently, Asif et al. taking advantage of this lack of data, evaluated three different doses (250 mg, 500 mg or 750 mg) of inhaled MgSO_4_ every 20 min for 60 min, in additive therapy to inhaled salbutamol. Children of either gender between two to 12 years of age, with the diagnosis of asthma having Pediatric Respiratory Assessment Measure (PRAM) score > 4. The combination of salbutamol with higher doses of nebulized MgSO_4_ resulted in an greater clinical improvement.^7^

In addition, we are struck by the concern of some studies with the preparation of the isotonic MgSO_4_ solution to be used in nebulization. The process has involved tonicity adjustments, pH control and sterilization, and dispensing in individual flaconettes (with different concentrations) by the pharmacists involved in the process. Few studies have detailed the preparation of their inhaled MgSO_4_ solutions.^6^

We can't ignore the fact that MgSO_4_ itself, administered intravenously, took a long time to be considered a safe and effective therapy. Until recently, some countries still had low adherence to this therapeutic option. Many studies carried out in adult and child populations have brought new data that has been used to justify the use of the intravenous route in care protocols for patients hospitalized for acute asthma.

We therefore agree with the observations made by Debiazzi et al. on the need for more studies, with adequate sample sizes and longer evaluation periods.^1^ However, it is important that methodological criteria which include dose distinctions and adequate preparation of inhaled MgSO_4_ solutions are also considered.

## Declaration of competing interest

The author declares no conflicts of interest.
